# American Text-Book of Gynecology

**Published:** 1894-03

**Authors:** 


### Book Reviews.

---

American Text-Book of Gynecology, 1893. W. B. Saunders & Co., Philadelphia, Pa. Price, Cloth, $6.00; Sheep, $7.00; Half Russia, $8.00. By subscription only.

Gives much attention to the important subject asepticand antiseptic hands, and instruments, the care in preparing the field of operation. No matter how familiar a person may be with this subject, he will find interest and benefit in this chapter. Chapters on Menstruation and its Anomalies, also Genital Tuberculosis, are well written and fairly presented, but sterility is dismissed with a passing notice.

The inflammatory diseases of the uterus, appendages and pelvic inflammation receive extended and careful consideration, such as these great and common subjects should. The pathology of the same is carefully and ably presented. Parts devoted to disease of the bladder, urethra and ureters possess great interest and much valuable matter to the reader.

The method of examination by Kelly is given in detail and so beautifully that one is possessed with a desire to order at once a set of instruments, and wonders how he managed to succeed so long without this method of examination.

The volume is profusely illustrated. Many new and important operations are there presented in a way that is easy of comprehension.

It is a valuable volume and remarkably free from glaring faults.

